# The effect of peer-group size on the delivery of feedback in basic life support refresher training: a cluster randomized controlled trial

**DOI:** 10.1186/s12909-016-0682-5

**Published:** 2016-07-04

**Authors:** Youngsuk Cho, Sangmo Je, Yoo Sang Yoon, Hye Rin Roh, Chulho Chang, Hyunggoo Kang, Taeho Lim

**Affiliations:** Department of Emergency Medicine, Hallym University Kangdong Sacred Heart Hospital, Seoul, Republic of Korea; Department of Emergency Medicine, Cha University Bundang Medical Center, 59 Yatap-ro, Bundang-gu, Seongnam-si, 463-712 Gyeonggi-do South Korea; Department of Emergency Medicine, Inje University College of Medicine, Busan, Republic of Korea; Department of Medical Education, Inje University College of Medicine, Busan, Republic of Korea; Department of Anesthesiology and Pain Medicine, Yonsei University College of Medicine, Seoul, Republic of Korea; Anesthesia and Pain Research Institute, Yonsei University College of Medicine, Seoul, Republic of Korea; Department of Emergency Medicine, Hanyang University College of Medicine, Seoul, Republic of Korea

**Keywords:** Cardiac arrest, Cardiopulmonary resuscitation, Basic life support, Training, Instructor, Medical education, Undergraduate medical education, Educational assessment, Feedback

## Abstract

**Background:**

Students are largely providing feedback to one another when instructor facilitates peer feedback rather than teaching in group training. The number of students in a group affect the learning of students in the group training. We aimed to investigate whether a larger group size increases students’ test scores on a post-training test with peer feedback facilitated by instructor after video-guided basic life support (BLS) refresher training. Students’ one-rescuer adult BLS skills were assessed by a 2-min checklist-based test 1 year after the initial training.

**Methods:**

A cluster randomized controlled trial was conducted to evaluate the effect of student number in a group on BLS refresher training. Participants included 115 final-year medical students undergoing their emergency medicine clerkship. The median number of students was 8 in the large groups and 4 in the standard group. The primary outcome was to examine group differences in post-training test scores after video-guided BLS training. Secondary outcomes included the feedback time, number of feedback topics, and results of end-of-training evaluation questionnaires.

**Results:**

Scores on the post-training test increased over three consecutive tests with instructor-led peer feedback, but not differ between large and standard groups. The feedback time was longer and number of feedback topics generated by students were higher in standard groups compared to large groups on the first and second tests. The end-of-training questionnaire revealed that the students in large groups preferred the smaller group size compared to their actual group size.

**Conclusions:**

In this BLS refresher training, the instructor-led group feedback increased the test score after tutorial video-guided BLS learning, irrespective of the group size. A smaller group size allowed more participations in peer feedback.

**Electronic supplementary material:**

The online version of this article (doi:10.1186/s12909-016-0682-5) contains supplementary material, which is available to authorized users.

## Background

The group size for BLS training is flexible, and there is currently no evidence-based recommendation for ideal student-instructor ratio. The European Resuscitation Council (ERC) recommends six students per instructor for the BLS Automated External Defibrillation Provider Course [1]. The American Heart Association (AHA) uses the same recommended ratio of six students to one instructor for their BLS course [2]. The recommended group sizes for ERC/AHA BLS courses are for the instructor-led training. However, the group size affect differently in the instructor-facilitated training when the instructor mainly facilitates peer feedback in the group.

The number of students in group feedback could affect students’ learning in different ways. A larger size of group permits intermittent rather than constant feedback to the students from instructor, but in turn, students have more opportunities to engage in peer facilitation. In a study that involved teaching manual chiropractic skills, intermittent feedback resulted in the best learning on acquisition and retention trials, while constant instructor feedback resulted in the most accurate acquisition of the manual skill on initial hands-on practice. The constant feedback was beneficial when used to reduce error during practice but detrimental when relied upon for retention and learning [3].

A systematic review identified feedback and repeated practice as the two most important features of simulation-based medical education [4]. Feedback after pre-training evaluation was also shown an effective method to improve BLS skill acquisition in two studies [5, 6]. Some BLS course using short video requires a facilitator rather than an instructor [7, 8]. Group discussion using video-based debriefing was another method to make better results in a BLS renewal training [9]. Two literatures suggest that peer assisted BLS training are easy to get feedback, more friendly and effective as expert instruction [10, 11]. However, it is not yet clear which group size is optimal on the delivery of feedback for learning BLS skills.

In the present study, we created an educational intervention that combined post-training test and agenda-led, outcome-based feedback to improve medical students’ BLS skills. The ‘agenda-led, outcome-based technique’ for feedback was originally described by Silverman et al. [12]. This method focuses on early acknowledgement of difficulties, removes defensiveness and anxiety about negative feedback, and allows discussion to improve students’ learning and enhance future performance on assessed tasks [13]. More instructor involvement might be necessary only when groups show little initiative, require help to analyse the experience at a deeper level, or demonstrate an inaptitude in independent discussion [14]. We aimed to investigate the effect of peer-group size on competency-based skills assessment in medical students undergoing a structured BLS refresher training.

## Methods

### Study design

A single-blind cluster randomized controlled trial was conducted on final-year (6th year) undergraduate medical students completing BLS refresher training during their emergency medicine (EM) clerkships. We provided simulator-based BLS training to 117 final year medical students. The training took place at the Medical Education and Simulation Centre at Hanyang University. Two students withdrew from the study due to personal reasons. Other eligible 115 students chose to participate in the study.

### Study setting and population

First, at the University, the final-year students were divided into 14 clinical rotation groups of approximately equal size. A representative of the Bureau of Students Affaires, blind to the study, conducted the group allocation of students. At the start of the emergency clerkship, the 14 clinical rotation groups were randomly assigned, through a sealed-envelope method, to one of two groups: standard or high student-instructor ratio. Standard ratio groups were divided in half and being trained separately. As a result, 58 students were allocated into 14 standard-ratio groups of 3–5 students and 57 students were allocated into 7 high-ratio groups of 7–10 students.

### Study protocol

**Fig. 1:**
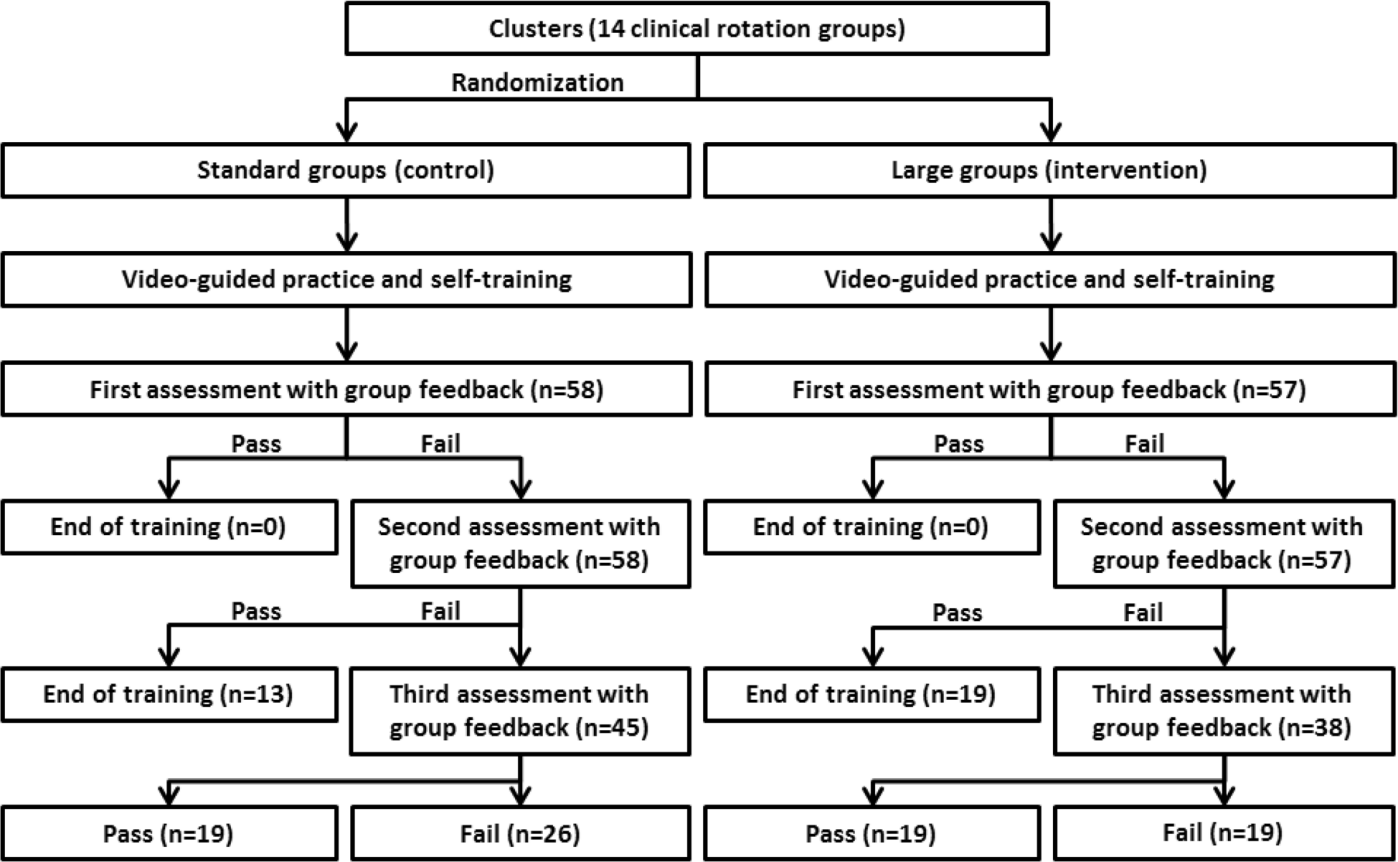
The participation flow chart

The training of one-rescuer adult BLS began with the guidance of an American Heart Association (AHA) certified BLS DVD (2010) designed for healthcare providers without any feedback. Subsequently, students participated in additional self-training using a cardiopulmonary resuscitation (CPR) manikin (Resusci Anne®, Laerdal, Norway). Group feedback was given after each student completed a post-training test, one at a time, in the presence of group members. Students who earned a perfect score finished their training after the first round of group feedback. Those who did not achieve a perfect score were offered additional group feedback. We designed the repeated post-training test with group feedback to allow students to master the BLS skills. We offered up to three sessions of group feedback due to course management difficulties. The tests and group feedback was videotaped for further analysis. The participant flow chart is shown in Fig. 1.

We used a predetermined observation checklist as a standard measure for test assessment and feedback. Instructors scored students’ performance live in the training session and gave them the assessed checklist. The group feedback was student’s agenda led and based on each assessment result. The instructor first asked the student independently what’s went wrong in their performance, and then encouraged the peer-group to brainstorm how to do better – not only to help the student but also to help themselves in their future practices. Each group was taught by one instructor accompanied by two researchers (YC, SJ) trained in the use of standard checklist.

### Measures

The primary objective was to examine group differences in post-training test scores. The results of each test were reported as a score between 0 and 100, with 100 as the passing score. The secondary outcomes included group differences in practice time, feedback time, number of feedback topics, and training evaluation questionnaire results. Video analysis was conducted to time each assessment. Practice time was defined as the time of a student’s BLS hands-on practice. Feedback time was defined as the period from the end of a student’s BLS hands-on practice to when the instructor confirmed group feedback topics were no longer being raised. Feedback topics were defined as the major themes of the group feedback that focused on the BLS assessment. The number of feedback topics was counted by how many topics were generated during each group feedback. At the completion of the study, students completed a questionnaire regarding their learning experiences. Four survey items addressed their attitudes towards the post-training test and group feedback. Other items asked for their opinion on the ideal number of students to instructor during BLS training and ideal number of BLS practices.

The checklist has 12 dichotomous items relating to the following procedures: check patient responses and call help (3 items), chest compression (5 items), and mouth-to-mouth ventilation (4 items). Based on the ratings of ten previously recorded videos of simulated BLS training with medical students, the intra-class correlation coefficient (ICC) between the two researchers (YC, SJ) for the checklist was high (ICC = 0.83; 95 % CI, 0.75–0.88). The instrument was derived by two other researchers (HG and TL) who had been involved in BLS skill assessment of medical students for years, and the researchers YC and SJ watched videos just once for determining the interrater correlation. The final version of the scoring instrument is provided in Additional file 1: Table S1.

### Data analysis

Statistical analyses were performed using SPSS 12.0 (SPSS Inc., USA). The continuous variables were analysed using analysis of covariance (ANCOVA) between standard- and high-ratio groups. To control for differences in CPR experiences, number of BLS experiences using CPR manikin in a BLS training at the start of clinical rotation and number of real CPR experiences during students’ clinical rotation before EM clerkship were included as covariates in the analysis. In comparison number of feedback topics per group, which of instructor (YC, SJ) facilitated the peer-group training was included as a covariate. Outcomes on first, second and third tests were analysed using repeated measures analysis of variance (ANOVA). A chi-square test was used to examine categorical variables. Continuous variables were expressed according to means and 95 % confidential interval, while categorical variables were expressed in counts and percentages. A value of *p* < 0.05 was considered statistically significant.

## Results

One hundred and fifteen final-year medical students were included in the present study. Table 1 presents demographic data about the final year medical students. All students have no previous BLS training except an instructor led-BLS training at the start of their clinical rotation 1 year ago. Number of one-rescuer adult BLS practices using CPR manikin in the previous BLS training were different in standard and large groups (3.4 vs 4.8; 95 % confidence interval (CI) 2.7–4.1 vs 3.2–6.4, *p* = 0.049). Forty-eight students (41.7 %) reported real experiences of CPR in their clinical clerkship, but the reported number of CPR experiences were low in both standard and large groups (0.4 vs 1.0; 95 % CI 0.2–0.6 vs 0.7–1.3, *p* = 0.001). The mean number of group members was 4.1 (95 % CI 3.9–4.3) in standard groups and 8.1 (95 % CI 7.7–8.5) in large groups (Table 1).

**Table 1:** Baseline characteristics of study participants (*N* = 115)

Variables	Standard group (*n* = 58)	Large group (*n* = 57)	*p*-value
Sex, female	21 (36.2 %)	21 (36.8 %)	1.000
Age, years	27.8 (26.8–28.8)	27.4 (26.6–28.2)	0.333
Number of one-rescuer adult BLS practices using			
CPR manikin in a previous BLS training at the start of clinical rotation	3.4 (2.7–4.1)	4.8 (3.2–6.4)	0.049
Number of real CPR experiences during students’ clinical rotation before EM clerkship	0.4 (0.2–0.6)	1.0 (0.7–1.3)	0.001*
Number of students in training group	4.1 (3.9–4.3)	8.1 (7.7–8.5)	<0.001*

Standard group, Standard group of 3–5 students with one instructor; Large group, Large group of 7–10 students with one instructor

Data are presented as number (%) or mean (95 % confidential interval)

Abbreviations: *BLS* basic life support, *CPR* Cardiopulmonary resuscitation

*p < 0.05 based on Student’s *t*-test between standard and large groups

**Fig. 2:**
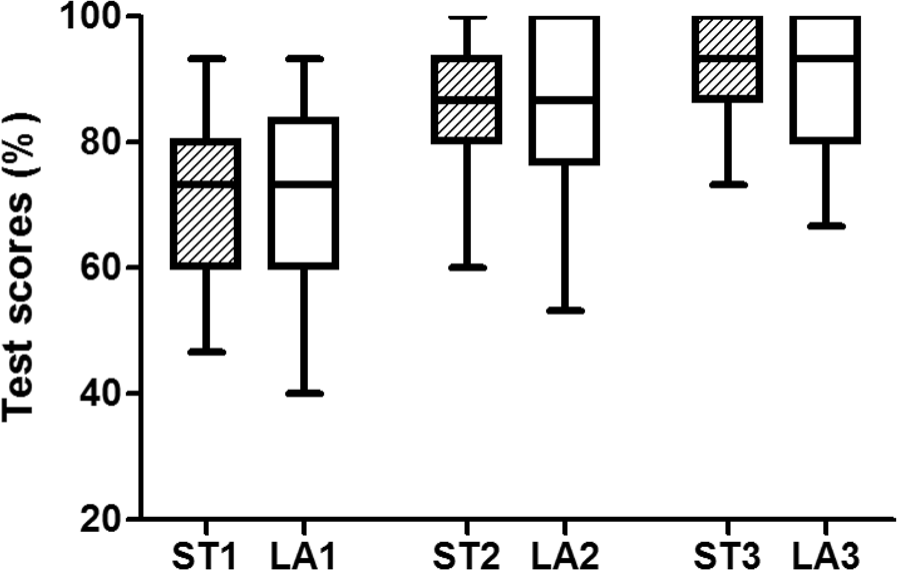
The scores between standard and large groups over three consecutive post-training test. ST1, scores in standard groups at first test;, LA1, scores in large groups at first test; ST2, scores in standard groups at second test; LA2, scores in high groups at second test; ST3, scores in standard groups at third test; LA3, scores in high groups at third test

The first test scores were low in both standard and large groups (72.2 vs 71.6; 95 % CI 68.5–76.9 vs 66.9–76.3), and statistically not significant between groups (*p* = 0.776). The second and third test scores with group feedback significantly increased in both standard (Second vs Third scores; 86.2 vs 93.8; 95 % CI 82.8–89.6 vs 91.3–96.3, *p* < 0.001) and large groups (Second vs Third scores; 86.2 vs 91.6; 95 % CI 81.9–90.5 vs 87.4–95.8, *p* < 0.001) (Table 2). However, the test scores did not differ between standard and large groups over the second and third post-training tests. *P*-values were *p* = 0.452 and 0.180, respectively (Fig. 2).

**Table 2:** Scores on each test in the BLS training between standard and large groups

Scores	Standard group	Large group	*p*-value^†^
First post-training test	72.2 (68.5–76.9)	71.6 (66.9–76.3)	0.776
Second post-training test	86.2 (82.8–89.6)	86.2 (81.9–90.5)	0.452
Third post-training test	93.8 (91.3–96.3)	91.6 (87.4–95.8)	0.180
*p*-value	<0.001^†^	<0.001^†^	-

Data are presented as mean (95 % confidential interval)

Standard group, Standard group of 3–5 students with one instructor; Large group, Large group of 7–10 students with one instructor

*p < 0.05 based on ANCOVA test between standard and large groups

^†^ *p* < 0.05 based on Repeated Measures ANOVA test between the first, second and third post-training tests

Video analyses showed that the practice time were not different between standard and large group participants, and were not changed during three consecutive tests. The feedback time were significantly higher in standard groups compared to large groups in the first test (241.7 vs181.1 s; 95 % CI 194.1–289.3 vs 139.9–222.3, *p* = 0.028) and the second test (126.1 vs 102.2 s; 95 % CI 97.3–154.9 vs 77.1–127.2, p = 0.026). The topic numbers discussed in the feedback time were also significantly higher in standard group participants compared to large group participants in the first test (2.8 vs 1.6; 95 % CI 2.2–3.4 vs 1.1–2.1, *p* < 0.001) and the second test (0.8 vs 0.5; 95 % CI 0.5–1.1 vs 0.3–0.7, *p* = 0.024). The statistical significance between standard and large group participants disappeared at the third test in both the feedback time (99.3 vs 117.6 s; 95 % CI 75.0–123.6 vs 66.6–168.7, 0.416) and the topic numbers (0.4 vs 0.4; 95 % CI 0.2–0.6 vs 0.1–0.7, *p* = 0.801). The total numbers of feedback topics in a peer-group were similar between standard and large groups in the three consecutive tests (Table 3).

**Table 3:** Practice time, feedback time, numbers of feedback topics on each test in the BLS training between standard and large groups

	Standard group	Large group	*p*-value^†^
Practice time (sec) per student			
First post-training test	185.1 (174.0–196.2)	177.2 (168.6–185.9)	0.231
Second post-training test	183.0 (174.6–191.4)	175.1 (167.5–182.7)	0.066
Third post-training test	182.4 (172.3–192.5)	178.9 (172.2–185.6)	0.508
*p*-value	0.490^†^	0.948^†^	-
Feedback time (sec) per student			
First post-training test	241.7 (194.1–289.3)	181.1 (139.9–222.3)	0.028*
Second post-training test	126.1 (97.3–154.9)	102.2 (77.1–127.2)	0.026*
Third post-training test	99.3 (75.0–123.6)	117.6 (66.6–168.7)	0.416
*p*-value	<0.001^†^	<0.040^†^	-
Number of feedback topics per student			
First post-training test	2.8 (2.2–3.4)	1.6 (1.1–2.1)	<0.001*
Second post-training test	0.8 (0.5–1.1)	0.5 (0.3–0.7	0.024*
Third post-training test	0.4 (0.2–0.6)	0.4 (0.1–0.7)	0.801
*p*-value	<0.001^†^	<0.001^†^	-
Number of feedback topics per group			
First post-training test	11.8 (8.8–14.8)	13.3 (7.9–18.7)	0.477
Second post-training test	3.1 (1.7–4.5)	4.0 (1.0–7.0)	0.457
Third post-training test	1.3 (0.5–2.1)	2.3 (0.2–4.4)	0.153
*p*-value	<0.001^†^	<0.001^†^	-

Data are presented as mean (95 % confidential interval)

Standard group, Standard group of 3–5 students with one instructor; Large group, Large group of 7–10 students with one instructor

*p < 0.05 based on ANCOVA test between standard and large groups

^†^p < 0.05 based on Repeated Measures ANOVA test between the first, second and third post-training tests

Students’ responses from the questionnaires in both standard and large groups showed that they thought the group feedback was helpful for their BLS training (4.98 vs 4.95; 95 % CI 4.94–5.00 vs 4.87–5.00, *p* = 0.140), and that they would recommend it to colleagues (4.72 vs 4.84; 95 % CI 4.45–4.99 vs 4.68–5.00, *p* = 0.362). Not only the group feedback for their actions (4.74 vs 4.91; 95 % CI 4.56–4.92 vs 4.79–5.00, *p* = 0.011), it is also helpful to discuss the performance of other students (4.76 vs 4.82; 95 % CI 4.50–5.00 vs 4.67–4.97, *p* = 0.679). The standard group participants recommended 4.4 (95 % CI 4.16–4.64) is the ideal group size, similar to their actual group size of 4.1 (95 % CI 3.9–4.3), but the large group participants wanted smaller group size of 5.8 (95 % CI 5.26–6.34) than their actual group size of 8.1 (95 % CI 7.7–8.5). For the ideal number of opportunities to practice one-rescuer adult BLS using CPR manikin in the training, the standard group participants wanted more opportunities compared to the large group participants (9.5 vs 5.8; 95 % CI 4.47–14.53 vs 4.71–6.89, *p* = 0.007), despite the actual number of practice were similar between groups (4.7 vs 4.6; 95 % CI 3.52–5.88 vs 3.51–5.69, *p* = 0.788) (Table 4).

**Table 4:** Questionnaire responses of 115 final-year medical students after the BLS training

	Standard group	Large group	P value†
Likert-type items (1–5), 1 = awful and 5 = excellent.			
Satisfaction of the teaching method	4.98 (4.94–5.00)	4.95 (4.87–5.00)	0.140
Recommendable to my colleagues	4.72 (4.45–4.99)	4.84 (4.68–5.00)	0.362
Feedback of my performance were helpful for my training	4.74 (4.56–4.92)	4.91 (4.79–5.00)	0.011*
Feedback of other students were helpful for my training	4.76 (4.50–5.00)	4.82 (4.67–4.97)	0.679
Non Likert-type items			
Recommendation for the ideal number of students to one instructor for the training	4.4 (4.16–4.64)	5.8 (5.26–6.34)	<0.001*
Recommendation for the ideal number of opportunities to practice one-rescuer adult BLS using CPR manikin in the training	9.5 (4.47–14.53)	5.8 (4.71–6.89)	0.070
Actual number of BLS practiced in the course	4.7 (3.52–5.88)	4.6 (3.51–5.69)	0.788

Data are presented as mean (95 % confidential interval)

Standard group, Standard group of 3–5 students with one instructor; Large group, Large group of 7–10 students with one instructor

*p < 0.05 based on ANCOVA test between standard and large groups

We analysed which BLS skills generated more feedback topics via video analysis. The five most popular discussion items are shown in Table 5. The scores of well-discussed items increased significantly over the post-training tests. Details regarding correct responses to each item on the checklist are described in Additional file 1: Table S1.

**Table 5:** Top five common topics discussed in the group feedbacks

1. Exact posture for carotid pulse check (16.2 %)
2. Exact posture of chest compression (12.6 %)
3. Exact position of chest compression when using manikin skill reporter (10.1 %)
4. How to call help (7.9 %)
5. Effective mouth-to-mouth ventilation when using manikin skill reporter (7.7 %)

## Discussion

The purpose of this study was to assess the effect of peer-group size on one-rescuer adult BLS skills assessment after the video-guided self-learning. Group feedback helps students find mistakes and learn other students’ coping strategies. It minimizes unnecessary time spent on correcting individual’s similar mistakes and less interruption may occur. Group feedback also provides trainees an opportunity to learn from each other and may even increase competition among students, which could stimulate students’ enthusiasm to learn and improve the quality and efficacy of training [15]. We assessed standard groups of 3–5 students with one instructor versus large groups of 7–10 students with one instructor.

In this study, the scores over three consecutive assessments showed no difference between the groups. Large group participants had more practices both in a previous BLS training at the start of their clinical rotation and in real CPR situations during their clerkship. We performed ANCOVA to control the differences with two covariates, but the measured scores between groups were not significantly different regardless of the two covariates “number of BLS hands-on practice in a previous BLS training and the number of real CPR experiences in the students’ clinical rotations”. A mean difference of 1.4 practices using manikin (standard vs large groups; 3.4 vs 4.8; *p* = 0.049) and of 0.6 real CPR experiences in clinical rotation (standard vs large groups; 0.4 vs 1.0, *p* = 0.001) might not advance medical students’ BLS skills.

The group size did not influence the assessment scores between groups in the present study. However, smaller group size provides greater participation in a BLS peer-group learning environment with a facilitator present. The standard groups made more feedbacks and requested more feedback time per students compared to large groups. The large group students selected more observation than direct participation. As much as direct learning via greater participation and more feedback for themselves, indirect learning such as observation and feedback for their peers could help the acquisition of BLS skills for the final-year medical students. In questionnaire results, most students answered feedbacks regarding other students’ performance were helpful. To the question about ideal group size, large group participants recommended smaller group size of 5.8 than their actual group size of 8.1. Interestingly, the standard group participants suggested the ideal group size was similar to their actual group size. For hands-on practice, standard group participants wanted more opportunities even though the number of hands-on practices were similar between groups. We suspect that the standard group size may benefit students’ willingness to participate in the learning of the BLS skills.

In peer-group feedback, peers directed the topic of feedbacks based on the scored students’ checklist. Students selected different strategies in peer-group facilitation. When the smaller group size provides greater participation, the students in large groups selected more observation than direct participation. However, total number of feedbacks in a peer-group were similar in both standard and large groups. Peer-group discussion centred on predefined checklists, thus the scope of feedbacks were not much different by group size. Even the groups had various size, the students had come close to master the BLS skills as its feedback topics decreased over the repeated training.

Basic life support (BLS) training using short video with a personal manikin is considered an effective alternative to instructor-led training [15, 16]. It is a convenient, inexpensive, and less time-consuming method than instructor-led training. However, some studies contend that video training with a personal manikin is inferior to instructor-led training [17]. In our study, the first test scores after the video-guided self-learning were low even they had already received BLS training in the previous year and had completed 1-year clinical rotations in specialties, such as General Surgery, Internal Medicine, Paediatrics, and Obstetrics/Gynaecology. Several students performed at a mediocre level and did not advance beyond self-validation. Individualised monitoring and feedback may counter limited improvement by providing challenges outside learners’ comfort zones as opportunities for deliberate practice [18]. Other multidisciplinary research on expert performance suggests that without adequate feedback efficient learning is impossible even among highly motivated subjects [19].

The percentage of correct answers on each item improved over the three consecutive post-training tests. Improvements were significant in ten of twelve items. The two unimproved items were ‘exact depth of chest compression’ and ‘exact chest recoil’. They were measured using a CPR manikin with a skill reporter and were not highly discussed in group feedback, even though their scores were low. We might use this identification of learning difficulties as a new starting point from which to improve future BLS training (e.g. sub-skill stations for high quality chest compression and effective mouth-to-mouth ventilation). Instructors should use assessment not only to measure a learner’s progress but also to acquire useful data to improve their own teaching practice [20].

Some limitations of our study should be mentioned. First, this study was conducted for the 4th-year medical students in a BLS refresher training during their EM clerkship 1 year after the initial training. Though we clustered the students following predetermined clinical rotation groups, we could not randomize the differences of CPR experiences between groups. Even we performed analysis of covariance to control the differences with covariates, unknown confounders may remain in the process of group allocation. Second, though most of the final-year medical students participated in this study, the statistical power for the primary outcomes was not enough. In a post hoc power analysis, this study require a sample size of 130 to detect a mean difference of 8.33 in test scores which the score of one item out of 12 checklist items, when we set the alpha level at 0.05 and the power at 95 %. We also are not certain whether the two groups had significant difference less than a score of one item in the test due to limited sample size. Third, this study assessed only the leaning on acquisition of BLS skills. Group size might affect differently on retention trials because smaller group students were willing to participate more BLS training. Fourth, this study was conducted in a medical school. Further research is needed to generalize our findings to other groups in different training environments. Fifth, we explored a restricted competence in BLS. Other important skills, such as using bag valve mask ventilation, an automated external defibrillation, and a two-person BLS method, were not evaluated in this study. Lastly, the two instructors could not be blind to the experiment because the number of group members was different. Even though we tried to standardize the assessment and feedback by using a pre-established checklist, personal bias could not be avoided.

## Conclusions

This study aimed to evaluate the influence of peer-group size on final-year medical students BLS training scores following video-guided BLS training. The group size did not impact the score of medical students between standard and large groups, but the BLS training scores increased after group feedback was repeated regardless of group size. The students participated more actively when the group was smaller and preferred a smaller group size. Therefore, this peer-group feedback approach after video-guided self-learning may improve students’ skill acquisition in BLS training and, thus, help them achieve predefined learning objectives.

## Abbreviations

AHA, American Heart Association; ANCOVA, analysis of covariance; ANOVA, analysis of variance; BLS, basic life support; CI, confidence interval; CPR, cardiopulmonary resuscitation; EM, emergency medicine; ERC, European Resuscitation Council; ICC, intra-class correlation coefficient

## Additional files

Additional file 1: Table S1. Scoring checklist and the percentage of correct answers per item. The checklist has 12 dichotomous items relating 3 categories: check patient responses and call help, chest compression, and mouth-to-mouth ventilation. (DOC 48 kb)

## References

[1] ERC Course Rules. European Resuscitation Council Website. https://faq.erc.edu/docs/erc_course_rules_VF20150320.pdf. Accessed May

[2] Providers Classroom Course & Materials: Frequently Asked Questions. American Heart Association Website. http://www.heart.org/idc/groups/heart-public/@wcm/@ecc/documents/downloadable/ucm_427486.pdf. Accessed 10 May 2015.

[3] Pringle RK (2004). Guidance hypothesis with verbal feedback in learning a palpation skill. J Manipulative Physiol Ther.

[4] Issenberg SB, McGaghie WC, Petrusa ER, Lee Gordon D, Scalese RJ (2005). Features and uses of high-fidelity medical simulations that lead to effective learning: a BEME systematic review. Med Teach.

[5] Li Q, Ma EL, Liu J, Fang LQ, Xia T (2011). Pre-training evaluation and feedback improve medical students’ skills in basic life support. Med Teach.

[6] Beckers SK, Biermann H, Sopka S, Skorning M, Brokmann JC, Heussen N (2012). Influence of pre-course assessment using an emotionally activating stimulus with feedback: a pilot study in teaching Basic Life Support. Resuscitation.

[7] Mpotos N, Lemoyne S, Calle PA, Deschepper E, Valcke M, Monsieurs KG (2011). Combining video instruction followed by voice feedback in a self-learning station for acquisition of Basic Life Support skills: a randomised non-inferiority trial. Resuscitation.

[8] Allan KS, Wong N, Aves T, Dorian P (2013). The benefits of a simplified method for CPR training of medical professionals: a randomized controlled study. Resuscitation.

[9] Na JU, Lee TR, Kang MJ, Shin TG, Sim MS, Jo IJ, Song KJ, Jeong YK (2014). Basic life support skill improvement with newly designed renewal programme: cluster randomised study of small-group-discussion method versus practice-while-watching method. Emerg Med J.

[10] Charlier N, Van Der Stock L, Iserbyt P (2016). Peer-assisted learning in cardiopulmonary resuscitation: the jigsaw model. J Emerg Med.

[11] Choi HS, Lee DH, Kim CW, Kim SE, Oh JH (2015). Peer-assisted learning to train high-school students to perform basic life-support. World J Emerg Med.

[12] Kurtz SM, Silverman JD, Draper J (1998). Teaching and learning communication skills in medicine.

[13] Carr S (2006). The foundation programme assessment tools: an opportunity to enhance feedback to trainees?. Postgrad Med J.

[14] Cleland J, Mackenzie RK, Ross S, Sinclair HK, Lee AJ (2010). A remedial intervention linked to a formative assessment is effective in terms of improving student performance in subsequent degree examinations. Med Teach.

[15] Bhanji F, Mancini ME, Sinz E, Rodgers DL, McNeil MA, Hoadley TA (2010). Part 16: education, implementation, and teams: 2010 American heart association guidelines for cardiopulmonary resuscitation and emergency cardiovascular care. Circulation.

[16] Soar J, Monsieurs KG, Ballance JH, Barelli A, Biarent D, Greif R (2010). Section 9. Principles of education in resuscitation: 2010 European resuscitation council guidelines for resuscitation. Resuscitation.

[17] de Vries W, Turner NM, Monsieurs KG, Bierens JJ, Koster RW (2010). Comparison of instructor-led automated external defibrillation training and three alternative DVD-based training methods. Resuscitation.

[18] Je S, Cho Y, Choi HJ, Kang B, Lim T, Kang H (2015). An Application the learning curve-cumulative summation test to evaluate the training of endotracheal intubation skill in emergency medicine. EMJ.

[19] Anders Ericcson K (2008). Deliberate practice and acquisition of expert performance: a general overview. Acad Emerg Med.

[20] Stiggins R (2004). New assessment beliefs for a new school mission. Phi Delta Kappan.

