# Supplementary material for: The effect of peer-group size on the delivery of feedback in basic life support refresher training: a cluster randomized controlled trial
Source: BMC Med Educ. 2016 Jul 4;16:167. doi: 10.1186/s12909-016-0682-5 (PMC4932763; doi:10.1186/s12909-016-0682-5)
Supplement: Additional file 1: Table S1. — Scoring checklist and the percentage of correct answers per item. The checklist has 12 dichotomous items relating 3 categories: check patient responses and call help, chest compression, and mouth-to-mouth ventilation. (DOC 48 kb) [file 12909_2016_682_MOESM1_ESM.doc]

<Table S1. Scoring checklist and the percentage of correct answers per item.

|  | Items | 1^st^ test  (N=115) | 2^nd^ test  (N=115) | 3^rd^ test  (N=83) | P value |
| --- | --- | --- | --- | --- | --- |
| 1 | Checks for response and for no breathing or only gasping (within 5 to 10 seconds)  -Tap the victim’s shoulder and shout “Are you all right?” | 78.3% (90) | 95.7% (110) | 97.6% (81) | <0.001* |
| 2 | Point a specific individual and call for help to activate EMS and get an AED | 73.0% (84) | 91.3% (105) | 98.8% (82) | <0.001* |
| 3 | Checks for carotid pulse (within 5 to 10 seconds) | 40.0% (46) | 76.5% (88) | 91.6% (76) | <0.001* |
| 4 | Place the heel of one hand on the centre of the victim’s bare chest (assessed by the skill reporting system during 5 cycles of CPR) | 72.2% (83) | 87.0% (100) | 91.6% (76) | 0.001* |
| 5 | Straighten arms and position shoulders directly over hands | 60.0% (69) | 77.4% (89) | 90.4% (75) | <0.001* |
| 6 | Compress chest at depth between 5 to 6 cm (assessed by the skill reporting system during 5 cycles of CPR) | 80.9% (93) | 88.7% (102) | 88.0% (73) | 0.19 |
| 7 | Compress chest at rate between 100 to 120 per minutes (assessed by the skill reporting system during 5 cycles of CPR) | 47.0% (54) | 78.3% (90) | 86.7% (72) | <0.001* |
| 8 | Allows complete chest recoil (assessed by the skill reporting system during 5 cycles of CPR) | 73.9% (85) | 75.7% (87) | 81.9% (68) | 0.40 |
| 9 | Open the victim’s airway with a head tilt-chin lift manoeuvre | 75.7% (87) | 87.8% (101) | 97.6% (81) | <0.001* |
| 10 | Give mouth-to-mouth breaths with visible chest rise (assessed by the skill reporting system during 5 cycles of CPR) | 22.6% (26) | 61.7% (71) | 74.7% (62) | <0.001* |
| 11 | Give 2 mouth-to-mouth breaths in less than 10 seconds | 80.0% (92) | 86.1% (99) | 95.2% (79) | 0.009* |
| 12 | Perform 5 cycles of 30 compressions and 2 breaths | 73.9% (85) | 85.2% (98) | 96.4% (80) | <0.001* |

EMS, emergency medical system; AED, automated external defibrillator; CPR, cardiopulmonary resuscitation.

Statistical differences of correct answers between each post-training test were calculated by the chi-square test.>
